# How Can Podiatrists and Other Health Care Professionals Support the Detection of Atrial Fibrillation?

**DOI:** 10.1002/jfa2.70043

**Published:** 2025-03-06

**Authors:** Jane E. A. Lewis, Joanna Tozer, Trudie Lobban, Andrea Evans, Matthew Banner, Lawrence Ambrose

**Affiliations:** ^1^ Cardiff Metropolitan University Cardiff UK; ^2^ Arrhythmia Alliance Warwickshire UK; ^3^ Hwyl Dda University Health Board Carmarthen UK; ^4^ Royal College of Podiatry London UK

**Keywords:** AF, atrial fibrillation, opportunistic detection, stroke and heart failure prevention

## Abstract

Atrial fibrillation (AF) is a global health crisis affecting 33.5 million people, with costs projected to reach £75 billion by 2035. A significant concern is that 43–48% of cases are asymptomatic, increasing the risk of stroke and heart failure. While general population screening lacks strong support, targeted screening shows promise in reducing stroke occurrence and healthcare costs. Podiatrists, who frequently treat adults of advancing age, are uniquely positioned to detect AF in high‐risk, asymptomatic individuals. This commentary advocates for opportunistic AF screening by podiatrists and other healthcare professionals, offering guidance for implementation. Early detection through defined referral pathways is crucial for timely diagnosis and management, potentially reducing AF‐related strokes that can lead to early mortality. Further high‐quality podiatry‐led studies are recommended to build on this commentary paper.

## 1 Background

**FIGURE 1:**
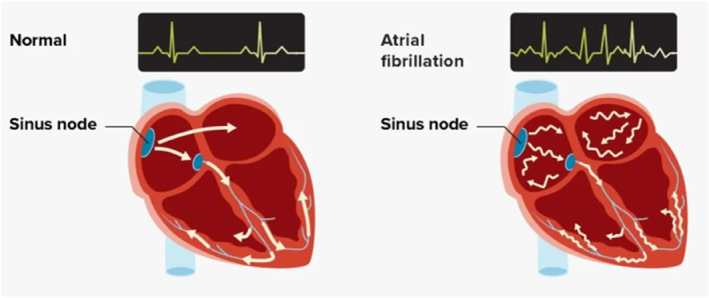
Normal sinus rhythm sends regular signals to each chamber. In AF, there are irregular signals sent from the heart’s atria. *Atrial fibrillation. Image credit: Alila Medical Media/Shutterstock*.

The frequency of atrial fibrillation (AF) diagnosis has increased to such an extent that it is now considered both an epidemic and a public health challenge [1]. AF occurs when the heart’s upper chambers (atria) experience chaotic electrical activity, causing them to quiver or fibrillate instead of contracting in a coordinated manner, disrupting the heart’s normal rhythm (Figure 1). The irregular contractions of the atria can allow blood to pool and form clots, which may then travel to the brain, potentially causing a stroke and increasing the risk of heart failure. Although AF in itself is not typically life‐threatening, it increases the risk of stroke and heart failure fivefold [2], highlighting the need for early detection and management. This commentary paper aims to support the role that community podiatrists and other healthcare professionals can play in conducting opportunistic AF screening.

AF has a current estimated prevalence of 33.5 million people worldwide [3], with estimated societal costs reaching £75 billion by 2035 [4]. Although most will present with symptoms, asymptomatic AF accounts for a substantial percentage of patients (43%–48%) and will likely go undetected and untreated [5].

The risk of developing AF increases with advancing age [6, 7, 8], as does the risk of developing hypertension, which accounts for approximately 1 in 5 cases of AF [9]. The prevalence of AF has been shown to increase with each decade of life beyond the age of 50 years, reaching almost 10% by the age of 80. Other risk factors include stress, all types of smoking and increased physical activity—for example, participating in endurance sports may lead to a higher risk of AF for some people, especially competitive athletes. At the same time, moderate physical activity can have a protective effect that can lower the risk of AF [10].

The differentiating features of abnormal heart rhythms are largely based on ECG findings and cardiovascular examinations. Table 1 provides a summary of differential diagnoses when considering heart arrhythmia.

**TABLE 1:** Differential diagnosis of heart arrhythmia.

Condition	Description
Atrial flutter or paroxysmal supraventricular tachycardia	When a short circuit in the heart causes the atria (upper chambers) to pump very rapidly for a short period of time (a few seconds or minutes). Indication for repeated checks.
Atrial tachycardia	When the heartbeat rises to > 100 beats a minute before returning to a typical heart rate of 60–80 beats a minute.
Atrioventricular nodal re‐entry tachycardia (AVNRT)	A type of abnormally fast heart rhythm that has an abrupt onset and termination.
Multifocal atrial tachycardia	A rapid irregular atrial rhythm that is caused by multiple ectopic foci (abnormal pacemaker sites) within the atria.
Wolff–Parkinson–White (WPW) syndrome	This is a relatively common heart condition that causes the heart to beat abnormally fast for periods of time. The cause is an extra electrical connection in the heart (accessory pathway). This problem with the heart is present at birth (congenital), although symptoms may not develop until later in life.

*Diagnosis of atrial fibrillation:* Although pulse palpation and confirmation with a 12‐lead ECG currently remain the gold standard for diagnosing abnormal heart rhythms [11], there has been an influx of mobile and wearable devices that have become available to the general population. Table 2 summarises the published evidence of the sensitivity and specificity of such devices when compared to the 12‐lead ECG [12].

**TABLE 2:** Sensitivity and specificity of various AF screening tools considering the 12‐lead ECG as the gold standard [12].

	Sensitivity	Specificity
Pulse taking	87%–97%	70%–81%
Automated BP monitors	93%–100%	86%–92%
Single lead ECG	94%–98%	76%–95%
Smartphone apps	91.5%–98.5%	91.4%–100%
Smart watches	97%–99%	83%–94%

Abbreviations: AF = atrial fibrillation; BP = blood pressure; ECG = electrocardiogram.

## 2 Indication for Asymptomatic Atrial Fibrillation Screening

There is little evidence to support general population screening; however, there is growing evidence in support of targeted asymptomatic screening. The European Society of Cardiology (ECS) Guidelines [12] describe the benefits of screening and finding asymptomatic AF, such as the prevention of subsequent onset of symptoms or AF‐related stroke, the prevention of AF‐related morbidity and the prevention of hospitalisation and mortality. A single‐lead ECG tracing of ≥ 30 s or a 12‐lead ECG showing AF, analysed by a medical professional with expertise in ECG rhythm interpretation, is fundamental in establishing a definitive diagnosis of AF [12]. Therefore, opportunistically screening those ≥ 65 years can lead to timely identification of asymptomatic AF. The Capture AF study [13], in 2020, explored a community pharmacy‐led atrial fibrillation detection referral service using the KardiaMobile AliveCor and PharmOutcomes national pharmacy database for their targeted opportunistic AF detection study. The quantitative outcomes highlighted the robust multidisciplinary referral pathway as their success. However, the cost‐effectiveness and health impact of the service were not reported. Savickas [14] explored the perspectives of those involved in the *Pharmacists detecting atrial fibrillation (PDAF) study* in a qualitative focus group study in Kent, the findings of which support the introduction of pharmacist‐led AF detection in general practice, highlighting the added value within an integrated service.

Because of increasing life expectancy, clinicians are encountering more AF in primary care, community health services and secondary care. As the population continues to increase in age, the number of individuals with AF is, therefore, set to increase, inevitably resulting in AF‐related stroke imposing a greater burden on patients, families and healthcare resources [15, 16]. It has recently been estimated that the stroke prevalence in the UK will rise from 950,000 in 2015 to almost 1.5 million in 2025 and over 2 million by 2035, an increase of 123% over the 20‐year period [17]. Opportunistic screening for asymptomatic AF potentially reduces AF‐related stroke occurrence, resulting in positive health outcomes and subsequent reductions in healthcare expenditure [18, 19, 20].

## 3 Guidance for Opportunistic Detection of Asymptomatic Atrial Fibrillation

Although we know that AF can occur at any age, we know that AF is particularly prevalent in the > 65‐year population [21], and we also know that AF is the most common and clinically relevant cardiac arrhythmia. Published estimates [1] are likely to be grossly underestimated due to the absence of routine opportunistic detection of asymptomatic cases, allowing for timely intervention. GP practices across the UK are experiencing significant and growing strain due to the decline in GP numbers, struggles to recruit and retain staff and rising demand, causing knock‐on effects for patients [22]. Consequently, this impacts the capacity for face‐to‐face GP appointments, which will only add to these underestimated figures.

Podiatrists will routinely be tasked with treating patients in the later stages of life [23]. As such, they are in a unique position to significantly contribute to the early detection of AF in high‐risk, potentially asymptomatic individuals, thus supporting this public health challenge to detect AF. Opportunistic screening can similarly be seamlessly integrated into standard examinations, leveraging the podiatrist’s existing practice of assessing foot pulses. It also creates the opportunity for patient education, giving patients the knowledge and skills to self‐monitor their pulses. The Arrhythmia Alliance provides resources such as ‘Know Your Pulse’ to support this education [24].

There have only been a handful of published podiatry‐led studies (Table 3) exploring the incidence of possible AF detection in podiatric practice.

**TABLE 3:** Summary of podiatry‐led published atrial fibrillation detection studies.

Author	Date	*N*	Target group	Duration	Number detected	Number confirmed	Change in management
Hicks et al. [25]	2019	5000	Diabetes mellitus (DM)	3 months	10	10	Not reported
Fisk and Lang [26]	2020	590	N/A	11 months	27	Stated that no follow‐up was conducted for confirmed cases	Not reported
Petridou et al. [27]	2021	300	DM	12 months	51	Not reported	Not reported

**FIGURE 2:**
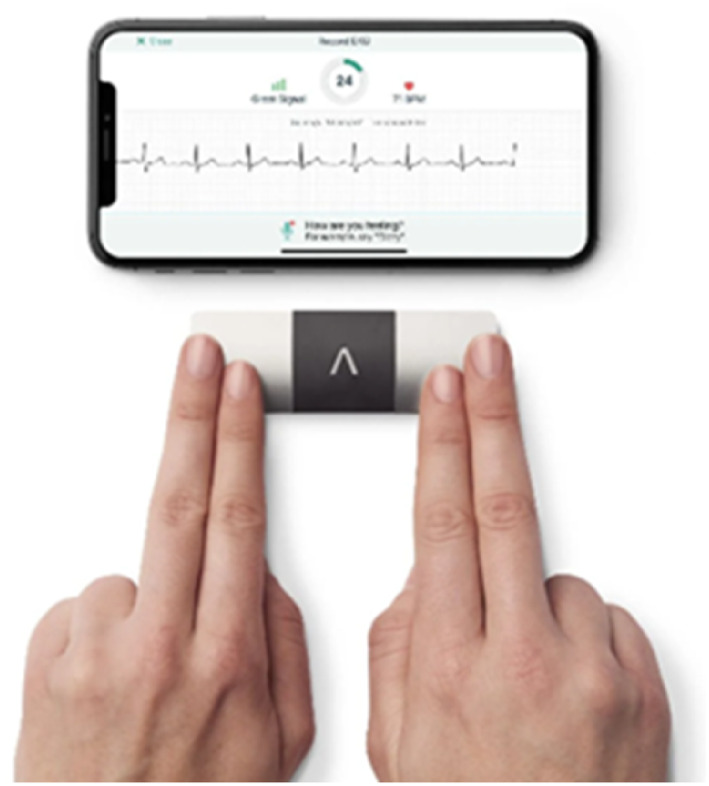
A demonstration of how to use the KardiaMobile AliveCor as an example of a 1‐lead ECG that is clinically validated for the detection of arrhythmias including atrial fibrillation*. Image credit: AliveCor.co.uk*.

Although pulse palpation and Doppler are the podiatrist's standard tools for detecting an arrhythmia, the KardiaMobile AliveCor (Figure 2) has clinical evidence to support its use as a 1‐lead or 6‐lead rhythm detection device in people with risk factors or symptoms of AF [28]. The AliveCor will also offer a PDF providing evidence to support any GP referral for confirmation with a 12‐lead ECG.

When AF is confirmed by a 12‐lead ECG, medical professionals recommend using the CHA_2_DS_2_‐VASc [29] for AF‐related stroke risk and QRISK3 [30] for the risk of heart failure as part of an AF risk assessment, alongside a detailed medical history, including reporting of any symptoms and asymptomatic abnormal pulse rhythm detected when considering someone's stroke risk and treatment planning [31]. Consideration of such tools may be useful when referring on to GPs.

Previous podiatry‐led studies [26, 27] have demonstrated the lack of follow‐up with GPs for outcomes once patients have been referred. Although simply asking the patient at their next appointment would seem sufficient, purposeful follow‐up will provide accurate outcomes of the 12‐lead ECG and any changes in medical management as a result of the podiatry‐triggered referral. For those with access, checking GP e‐records can facilitate this follow‐up.

## 4 Clinical Application

*Training and awareness:* With training, podiatrists and other healthcare professionals can learn to recognise abnormal heart rhythms when taking pulse readings of patients’ feet. Pedal pulses should be located using the index and middle fingers. Light contact should be maintained for at least 30 s to identify a regular or irregular rhythm. A handheld Doppler with coupling gel can be used in the same way to allow the healthcare professional to make a judgement on the audible pulse having a regular or irregular rhythm. This enhanced skill allows them to identify potential AF cases during any routine appointment. The possibility of paroxysmal AF (a short period of irregular heart rhythm) suggests that repeating pulse checks at every contact is recommended.

*Targeting high‐risk groups:* Podiatrists regularly see patients who are at higher risk for AF, such as adults of advancing age and those living with diabetes [23, 32]. By focusing screening efforts on these at‐risk populations during foot checks, podiatrists can help detect AF in those most likely to have the condition.

*Integration into existing workflows:* AF screening can be seamlessly integrated into existing podiatry appointments when considering vascular health, which makes it a cost‐effective and efficient method of opportunistic screening.

**FIGURE 3:**
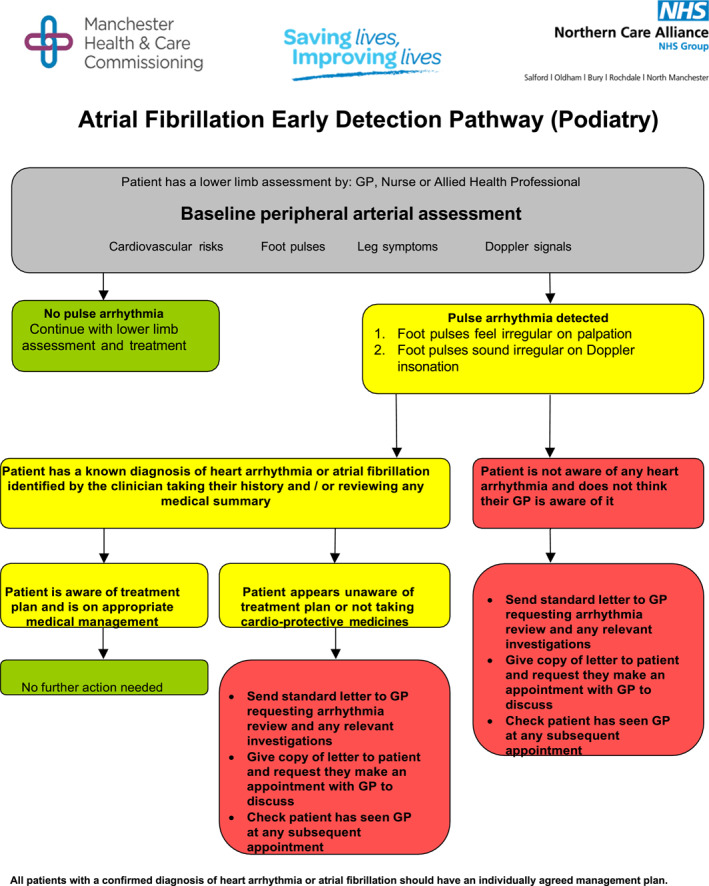
NHS Northern Care Alliance atrial fibrillation clinical pathway [33].

*Referral pathways:* When an irregular pulse is detected, podiatrists can refer patients for further cardiac evaluation and formal diagnosis. Establishing clear referral pathways is essential to ensure timely follow‐up and appropriate management of potential AF cases. The clinical pathway may vary slightly between localities; however, the Royal College of Podiatry has endorsed the guidance from the NHS Northern Care Alliance on such clinical pathways in their policy position on AF detection [33] (Figure 3). Table 4 provides a quick reference guide for all healthcare professionals, highlighting the key steps when screening for AF.

**TABLE 4:** A quick reference guide for atrial fibrillation detection.

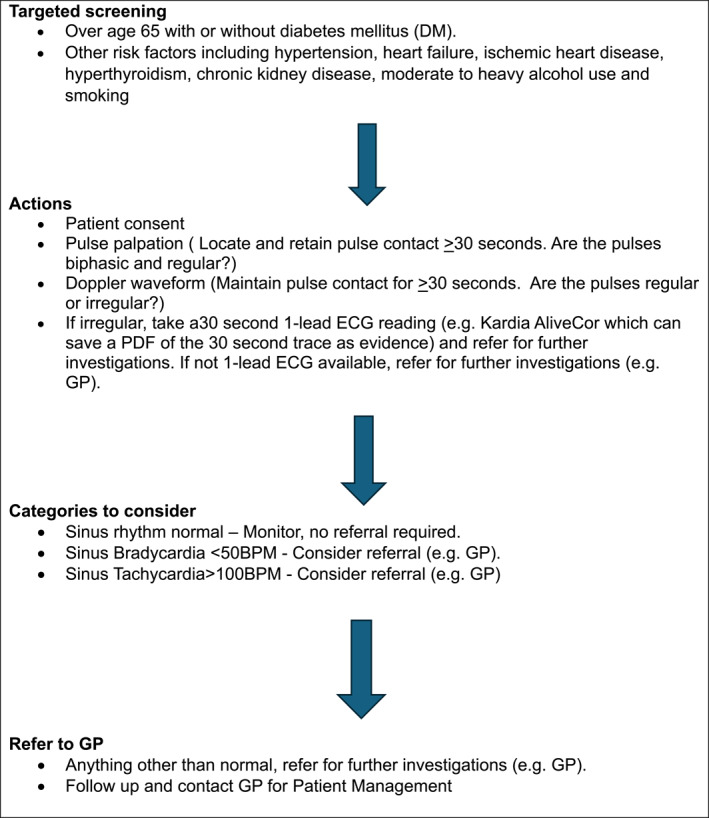

*Potential impact:* Studies [25, 26, 27] have shown promising results for this opportunistic approach; however, follow‐up to establish the number of confirmed AF cases and any changes in management was not considered by most. Follow‐up is essential to give a true reflection of the impact of such screening.

## 5 Limitations

Although pharmacy‐led AF detection studies have published evidence to support opportunistic AF detection within their practice [13, 14], this commentary paper focused on podiatry‐led AF detection studies due to the obvious pedal pulse vascular assessment nature of the profession. However, these appeared limited and lacked any rigour. As the authors did not perform a systematic review, they do acknowledge that they might have missed eligible or unpublished studies.

## 6 Conclusion

This paper makes a case in support of the role of community podiatrists and other healthcare professionals in conducting opportunistic AF screening. The paper refers to the modified pathway endorsed by the Royal College of Podiatry (Figure 3) but emphasises the critical need for a well‐defined, robust, locally agreed referral pathway [13]. Such a pathway facilitates timely access to 12‐lead ECG tests, formal diagnosis and appropriate management strategies, which are essential components in the effort to reduce AF‐related strokes and heart failure incidents.

Because of the lack of published evidence, the authors recommend that further high‐quality, podiatry‐led, opportunistic AF detection studies are required to provide robust evidence to support the adoption of opportunistic detection of AF by their own and other healthcare professionals. These studies should include robust research methods designs, include an agreed clinical referral pathway that can be evaluated and comment on the health and economic impact. The authors also recommend that a future systematic review is conducted in the field to build on this commentary paper.

## Conflicts of Interest

The authors declare no conflicts of interest.
